# 

**Published:** 1914-11-21

**Authors:** 


### Medical Appointments.

Dr. David Sommerville has been appointed medical officer of health for Harrow in succession to the late Dr. Fletcher Little.

The following appointments have been made at Manchester Children's Hospital: Miss Mary A. Kirk, M.B., Ch.B., to be senior resident medical officer; Miss Nora Hamill, M.R.C.S., L.R.C.P., to be junior resident medical officer; and Mr. H. F. Hutchinson, M.B., Ch.B., to be senior medical officer at the out-patients' department, Gartside Street, Manchester.

The Rev. Norman Shelton has been appointed to act as chaplain to Addenbrooke's Hospital, Cambridge, on the resignation of the Rev. S. T. Adams.

Dr. J. E. Payne, one of the honorary surgeons of the Torbay Hospital, has joined the staff of one of the hospitals in France for the wounded. The recently appointed house surgeon, Dr. H. A. Cookson, has accepted a commission in the R.A.M.C., and left last week.

### Gifts and Bequests.

Mr. James Grimble Groves, of Springbank, Pendleton, Salford, Manchester, Unionist M.P. for South Salford, 1900-6, has left £1,000 to the Salford Royal Hospital for the endowment of a "James Grimble Groves Bed," £1,000 to the Pendlebury Hospital for Children for the endowment of a "James Grimble Groves Cot," and £1,000 to the Altrincham Provident Hospital and Dispensary for the maintenance of the "Colin Cot" provided by him some time ago.

Recent legacies to the Leicester Royal Infirmary are £100 under the will of the late Miss E. K. Varnam, £20 under the will of the late Mr. Alfred Cooper, of Sewstern, and £10 10s. under the will of the late Mrs. Mary Olphin.

The Treasurer of Bradford Royal Infirmary has received a cheque for £50 from Mr. F. M. Hood, Thornleigh, Ilkley, in payment of a legacy of this amount bequeathed by his late father, Mr. B. M. Hood, of Eldermere, Ilkley.

The late Alderman G. Kett, of Cambridge, who was for many years a governor of Addenbrooke's Hospital and served on the old weekly board and afterwards on the general committee, taking a great interest in the institution, has by his will bequeathed the sum of £100 to the funds.

The authorities at Addenbrooke's Hospital, Cambridge, have received £12 18s. 6d. as a donation for the benefit of a child's cot in the hospital, collected by Miss Rosemary and Master Freddie Thornton, of Little Shelford. This is but another example of what valuable help can be rendered to our hospitals by the children.

Miss M. A. Clay, of West House, Cambridge, has sent the sum of £10 to the Building Fund of Addenbrooke's Hospital, Cambridge. Other subscribers to the Fund include Sir W. Grahame Green, K.C.B., of Harston, Cambridgeshire, who has sent a donation of £47 18s.; and, among legacies, is one of £100 from the late Alderman G. Kett, for many years a member of the weekly board.

The Chelsea Hospital for Women has received £30 9s. 3d. as the result of the collection made at the Palladium Theatre at the recent special matinée which was held in aid of the National Volunteer Reserve. The hospital is giving special care to the relations of men called to the front.

Mr. George Wilson, M.B., sometime of Edinburgh, and latterly medical missionary, of Hope House, Tangier, Morocco, left personal estate value £1,754.

### The Book Market.

LIST OF NEW BOOKS RECEIVED FROM THE FOLLOWING PUBLISHERS:—

J. Wright and Sons, Limited: "Obiter Scripta. Throat, Nose and Ear." By A. R. Friel, M.A., M.D. 2s. 6d. net. "Urgent Surgery." Vol. I. Third English Impression. By Felix Lejars. Translated by W. S. Dickie, F.R.C.S. 25s. net.

Macmillan & Co., Limited: "Chemistry for Nurses." By R. Ottenberg, A.M., M.D. 4s. 6d. net.

### Institutional and Public Reports.

Report (for 1913) of the Sub-Committee and of the Medical Superintendent of the West Riding Pauper Lunatic Asylum, Wakefield.

### Glasgow Hospitals and Municipal Control.

In the Note which appeared under the above heading in The Hospital for the 14th instant, page 145, the word "placed" in the thirteenth line in some portion of our edition was inadvertently inserted instead of "co-ordinated."

It is announced by the various societies for the relief of the sick and wounded that old sheets, unbleached calico, and old linen suitable for bandages are wanted, but not old clothes. Offers of food and invalid delicacies have been very numerous, but till the wounded become ready to be discharged as convalescents there seems to have been no realisation of the need for boots.

RATES OF SUBSCRIPTION: Twelve months, 6/6; Six months, 4/-; Three months, 2/-, post free to any address in the United Kingdom. Abroad, Twelve months, 8/8; Six months, 5/-; Three months, 2/6, post free.—Address The Subscription Dept., "THE HOSPITAL," The Hospital Building, 28/2 Southampton Street, Strand, London W.C.

